# Emerging Role of Histone Acetyltransferase in Stem Cells and Cancer

**DOI:** 10.1155/2018/8908751

**Published:** 2018-12-16

**Authors:** Daniela Trisciuoglio, Marta Di Martile, Donatella Del Bufalo

**Affiliations:** ^1^Institute of Molecular Biology and Pathology, National Research Council (CNR), Via Degli Apuli 4, Rome 00185, Italy; ^2^Preclinical Models and New Therapeutic Agents Unit, IRCCS-Regina Elena National Cancer Institute, Via Elio Chianesi 53, Rome 00144, Italy

## Abstract

Protein acetylation is one of the most important posttranslational modifications catalyzed by acetyltransferases and deacetylases, through the addition and removal of acetyl groups to lysine residues. Lysine acetylation can affect protein-nucleic acid or protein-protein interactions and protein localization, transport, stability, and activity. It regulates the function of a large variety of proteins, including histones, oncoproteins, tumor suppressors, and transcription factors, thus representing a crucial regulator of several biological processes with particular prominent roles in transcription and metabolism. Thus, it is unsurprising that alteration of protein acetylation is involved in human disease, including metabolic disorders and cancers. In this context, different hematological and solid tumors are characterized by deregulation of the protein acetylation pattern as a result of genetic or epigenetic changes. The imbalance between acetylation and deacetylation of histone or nonhistone proteins is also involved in the modulation of the self-renewal and differentiation ability of stem cells, including cancer stem cells. Here, we summarize a combination of *in vitro* and *in vivo* studies, undertaken on a set of acetyltransferases, and discuss the physiological and pathological roles of this class of enzymes. We also review the available data on the involvement of acetyltransferases in the regulation of stem cell renewal and differentiation in both normal and cancer cell population.

## 1. Introduction

Epigenetic changes do not involve changes in the DNA sequence but alter the physical structure of DNA. To date, the most commonly epigenetic changes include DNA methylation and histone modifications, such as methylation and acetylation at lysine residues. Lysine acetylation is catalyzed by lysine acetyltransferase, formerly called histone acetyltransferase (HAT), which transfers the acetyl group of acetyl-CoA to the epsilon-amino group of an internal lysine residue located near the amino termini of core histone proteins [1]. The reverse reaction is accomplished by deacetylases (HDAC). More recently, other posttranslational modifications of histones have been described such as neddylation, sumoylation, glycosylation, phosphorylation, poly-ADP ribosylation, and ubiquitination [2]. All these posttranslational modifications of histones, as well as nonhistone proteins, regulate gene expression profiles through their effect on chromatin structure/remodelling. Histone acetylation is associated with an open and active chromatin conformation (i.e., euchromatin), while histone deacetylation is generally associated with a condensed and inactive form of chromatin (i.e., heterochromatin). On the other hand, histone methylation might be a marker for both active chromatin and inactive chromatin.

For definition, it is not possible to pass down epigenetic changes to future generations; nevertheless, it is now accepted that epigenetic modifications can cross the border of generations and can be inherited from parent to offspring. In line with the relevance of epigenetic changes in normal development, the first stage of development is evidenced by erasure of epigenetic information compatible for development. This epigenetic phenomenon, named epigenetic reprogramming, is likely required for resetting the epigenome of the early embryo, so that it can form every kind of cell type in the organism. To pass to the next generation, epigenetic information must avoid being erased during reprogramming. Indeed, it is now well accepted that there are rare regulatory elements that evade, for instance, DNA demethylation during embryogenesis, thus suggesting that change in the epigenome can be inherited also transgenerationally [3–5]. In line with this evidence, two recent studies evidence that also maternal inheritance of histone marks trimethylated lysine 27 of histone 3, a repressing mark of gene expression, may represent a conserved mechanism able to regulate gene expression during early development [6, 7]. Overall, these studies recognize the importance of epigenetic programming in determining cell identity during the reprogramming process, indicating that epigenetic information might play a critical role in the restoration of totipotency in the embryo or in stem cells.

An aberrant epigenetic signature can be responsible for some disease states causing abnormal activation or silencing of genes playing a role in different pathologies, such as syndromes involving chromosomal instabilities or mental retardation [8, 9]. Epigenetic alterations can also be responsible for the promotion or inhibition of a malignant phenotype at various stages of the disease: in transformed cells, epigenetic changes occur in key oncogenes or tumor suppressor genes leading to cancer initiation or progression [10, 11].

The aim of this review is to discuss the role of protein acetylation leading to cancer initiation and progression, and their role in the maintenance of stem cell progenies and how deregulation of HAT in this subpopulation sustains tumor development.

## 2. HAT: Classification and Functions

Histone acetylation is preferentially carried out on specific lysine: for instance, histone H3 is mainly acetylated in positions 9, 14, 18, and 23, while the lysine of histone H4 that are preferentially acetylated are in positions 5, 8, 12, and 16. The addition of the acetyl group neutralizes the positive charge of lysine weakening the electrostatic interaction between the histones and DNA, relaxing the chromatin structure and recruiting chromatin remodelling protein complexes (e.g., transcription factors and chromatin modifiers), and finally leading to gene activation. Recent analysis of lysine acetylation through mass spectrometry has increased our understanding on this posttranslational modification [12] and demonstrated the involvement of HAT enzymes in many biological processes beyond gene transcription, through the regulation of protein interaction, activity and cellular localization. Thus, the human HAT have been recently renamed as lysine acetyltransferases (KAT), for their ability to acetylate different proteins beyond histones.

The main function shared by all HAT members is the activation of transcription. They are classified into type A and type B on the basis of their localization inside the cell (Table 1). Type A shows mainly nuclear localization, likely catalyzes the processes related to transcription, and is grouped into five main families:

1. p300/CBP
2. GCN5-related *N*-acetyltransferase (GNAT)
3. Moz, Ybf2/Sas3, Sas2, Tip60 (MYST)
4. Nuclear receptor coactivator- (NCOA-) related HAT
5. Transcription factor-related HAT

In the past years, the Camello family has also been included in this classification. The novel Camello HAT family has been identified in zebrafish and includes functional HAT showing specificity towards histone H4 and perinuclear localization [13]. Type B consists of HAT1, HAT2, HatB3.1, Rtt109, and HAT4 and it is localized in the cytoplasm.

The p300/CBP family consists of two members with similar structure and functions: CBP (CREB-binding protein) and its paralog p300. Both CBP and p300 contain an HAT domain of about 500 residues, in which they share 86% sequence identity, a bromodomain, and three cysteine-histidine-rich domains (TAZ, PHD, and ZZ) serving for the protein-protein interaction. CBP/p300 act as coactivators of hundreds of different transcription factors, and it is now well clear that they are key regulators in the assembly and mobilization of the basal transcription machinery [14]. In this context, it has been suggested that p300/CBP binding to transcription factor activation domains positions HAT near specific nucleosomes in target gene promoter regions thus facilitating the transcriptional activation [15]. It has also been reported that both p300 and CBP modulate the activity and cellular localization of different factors producing multiple downstream effects in the cells [16–18].

The GNAT family consists of at least 12 enzymes with different cellular functions that acetylate both histone and nonhistone proteins. They contain an HAT domain of around 160 residues and a conserved BRD at the carbossi-terminus, which recognizes and binds to acetyl-lysine residues [19]. The two main members, GCN5 (general control nonderepressible 5, KAT2A) and pCAF (p300/CBP-associated factor, KAT2B), are closely related proteins playing an important role in gene transcription. Beyond their gene-specific HAT activities, the GCN5 and pCAF enzymes have been shown to acetylate numerous transcription factors, thus regulating their functions [20]. Despite a cytosolic localization, *α*-tubulin acetyltransferase 1 (ATAT1), mainly responsible for *α*-tubulin acetylation at lysine 40 in higher organisms, has been included in the GNAT superfamily [21–24]. Acetylation of histone and nonhistone proteins by GNAT controls gene transcription, DNA replication, DNA repair, cell cycle progression, cell signalling pathways, and metabolism. GNAT enzymes are known to play a role in a wide range of human diseases including cancer, obesity, diabetes, and metabolic disease [25–27]. The MYST family comprises five enzymes: MOZ, Tip60, MOF, MORF, and HBO1. This family is characterized by the presence of a highly conserved 370-residue MYST domain and other domains relevant for the recognition of other proteins [28, 29]. Members of this family play a critical role in a wide range of cellular processes including regulation of transcription, cell growth, and cell cycle [28].

The nuclear receptor coactivators family includes steroid receptor coactivators (SCR1, SCR2, and SCR3). SCRs are coactivators that are required for transcriptional activity of the steroid receptor superfamily. SRC1 has an HAT domain at its carbossi-terminal region and is primarily specific for histones H3 and H4 [30], thus being involved in both chromatin remodelling and the process of recruitment/stabilization of general transcription factors [26]. The transcription factor-related HAT family includes TATA box-binding protein- (TBP-) associated factors TAFII250 and TFIIIC [31]. The most studied members of HAT of type B are HAT1 and HAT4 [32, 33]. In humans, both HAT promote the acetylation of cytosolic histone H4 favouring the nucleosome assembly, whereas HAT1 also acetylates histone H2A on lysine 5 [32–34].

## 3. Role of HAT in Development and Cancer

It is well clear that for their effect on gene transcription or on nonhistone proteins, HAT enzymes and consequently protein acetylation are implicated in development and physiology and in the genesis of several diseases. Thus, it is unsurprising that HAT are also involved in the regulation of stemness properties of normal and cancer cells.

### 3.1. Roles of HAT in Development and Physiology

Recent studies on HAT-null and heterozygous mice have revealed highly specific functions of individual enzyme in development, physiology, and disease (Table 2). Indeed, this is possible not only for the canonical function of HAT on gene transcription but also for their structural role as a scaffold protein.

The role of CBP/p300 in neural development has been described in several studies by using mutant mice for these HAT. Loss of both p300 and CBP results in early embryonic lethality. Moreover, the mutant embryos display several neural tube closure and embryonic vascular and cardiac defects. Notably, also heterozygous mice for p300 manifest considerable embryonic lethality. More recent studies using p300/CBP conditional knockouts reveal a distinct role for p300 and CBP in defined cell lineages, although both genes are essential for cell proliferation [35, 36]. Mice genetically deleted for CBP represent also a good model to study Rubinstein-Taybi syndrome (RTS), a cognitive disorder prominently linked to the deficiency in CBP activity [8]. Of note, mice with a mutant form of CBP, lacking its HAT domain (CBP^Δ^ mice) or with point mutations in the domain mediating the CREB interaction (CBP^KIX/KIX^ mice), showed some defects in memory and synaptic plasticity [37, 38].

Mice homozygous for mutation in the KIX domain of p300 showed multilineage defects during the hematopoiesis, such as B-cell deficiency, megakaryocytosis, and thrombocytosis, thus indicating that binding of p300 to c-Myb and CREB is required for hematopoiesis [39].

The early embryonic lethality was also observed in GCN5-null mice [40]: contrary to GCN5-null embryos, *GCN5*^hat/hat^ embryos, with point mutations that abrogate GCN5 HAT activity, are viable but show cranial neural tube closure defects and exencephaly [41]. Of note, the defects of GCN5-null mice are due not only to the effect of GCN5 on histone acetylation but also to the effect on other GCN5 target proteins. In line with this evidence, deletion of p53, a well-known GCN5 nonhistone target, partially rescued the defect of GCN5-null embryos [41].

On the contrary, loss of pCAF did not determine obvious abnormal phenotypes in mice [42, 43]. Despite this evidence, defects in learning abilities and both short-term memory and contextual long-term memory have been observed in adult pCAF-null mice [44]. A more recent paper described the defect associated with pCAF and GCN5 loss in zebrafish development. Indeed, morpholino-mediated knockdown of pCAF and GCN5 transcripts severely perturbs heart and limb development, and pharmacological inhibition of HAT also produces cardiac and fin defects during zebrafish development [45]. The *α*-tubulin acetyltransferase ATAT1 is expressed in both mouse embryos and tissues. ATAT1-null animals were viable, and no morphological defects were found, despite the fact that this acetylation of *α*-tubulin is lost in sperm flagella and the dentate gyrus is slightly deformed [46].

Homozygous mutations of the main MYST members also result in early embryonic lethality; in contrast, heterozygous mutations exhibit no relevant phenotypes.

### 3.2. Roles of HAT in Stem Cell Maintenance

Stem cells are defined as a class of undifferentiated cells that for definition (i) replicate indefinitely maintaining an undifferentiated state (or self-renewal capacity) and (ii) differentiate into specialized cell types (or cell potency). Commonly, stem cells are derived from two main sources: (i) embryos formed during the blastocyst phase of embryological development (embryonic stem cells (ESCs)) and (ii) adult tissues (somatic or adult stem cells). Both types are generally characterized by their potency to differentiate into different cell types [47]. ESCs exhibit the ability to avoid replicative senescence maintaining their undifferentiated state and to differentiate into any different specialized cells derived from the three germ layers (ectoderm, endoderm, and mesoderm). The main difference between embryonic and adult stem cells is the pluripotency, as adult stem cells are considered multipotent, namely, stem cells that are able to differentiate in a lineage-restricted manner. Adult stem cells are named on the basis of their tissue of origin (e.g., mesenchymal stem cell, endothelial stem cell, and dental pulp stem cell), and they act mainly as a repair system for the renewal of adult tissues. Using a genetic reprogramming, it is possible to obtain a type of pluripotent stem cell directly from adult cells [47]: these cells are named induced pluripotent stem cells (also known as iPS cells or iPSCs). They are very similar to ESCs and may represent an attractive approach for regenerative medicine.

The maintenance of stem cell properties requires the activation of a series of transcription factors, among them NANOG, OCT4, SOX2, KLF4, and c-Myc, while several signalling pathways, including LIF/STAT3, BMP, PI3K, FGF2, Wnt, TGF*β*, and MAPK pathways, and epigenetic factors, including HAT, HDAC, and DNA methyltransferases, play an important role in stem cell pluripotency reprogramming [48, 49].

Different evidences showed a regulatory mechanism indicating an acetylation-related effect on stemness, and several studies identified the specific HAT involved in the regulation of stemness property of normal stem cells (Table 3). In this context, a misregulation of HAT may lead to an altered potential of self-renewal and expansion of epigenetically modified stem cell pools [50].

Genome-wide and mass spectrometry experiments have demonstrated the lysine 56 acetylation (K56Ac) in histone H3 also in mammal cells. Notably, high levels of K56Ac mark the pluripotency transcriptional network in human ESCs and correlate positively with binding along promoters of OCT4, NANOG, and SOX2 [51]. Interestingly, in mouse ESCs, OCT4 interacts with H3 K56Ac. This interaction is likely direct and promotes the pluripotency of ESCs [52]. In another independent study, aimed at evaluating the levels of histone posttranslational modifications during the differentiation of ESCs, a global decrease in multiply acetylated histone H4 peptides was found [53], suggesting the relevance of this modification in the maintenance of stemness. Acetylation of histone H3K9, an epigenetic mark associated with open chromatin structures, is involved in the neural commitment from ESCs, and p300 has been identified as the enzyme involved in both ESC pluripotency and neural differentiation [49].

In an experimental model of iPS cells, p300 promotes acetylation of OCT4, SOX2, and KLF4 at multiple sites to change their transcription activity, thus regulating stem cell reprogramming [54]. In line with this evidence, p300 has been reported to regulate the expression of NANOG and SOX2 and the proliferation and odontogenic differentiation of human dental pulp cells, regulating the expression of key pluripotency genes [55, 56]. p300 and CBP also play redundant roles in maintaining the undifferentiated state of ESCs. Indeed, both are recruited by NANOG through the physical interaction to NANOG-binding loci, mediating the formation of p300/CBP-binding loop fragments containing enhancer activities, suggesting that the formation of these higher-order chromosome structures is important in maintaining self-renewal and pluripotency of ESCs [57].

Histone and nonhistone protein acetylation regulates also normal hematopoiesis [58, 59], being a network of epigenetic regulators, including NuA4/p300/CBP/HBO1, needed for normal and hematopoietic development [60]. p300 and CBP play essential but distinct roles in maintaining hematopoietic stem cell (HSC) self-renewal and regulating differentiation into committed hematopoietic progenitors. In particular, while CBP is relevant for HSC self-renewal, p300 is essential for hematopoietic differentiation [61].

GCN5 is essential for embryonic survival in mice [42, 43] and is highly expressed in mouse ESCs compared with differentiating cells [62]. Recently, GCN5 has been identified as a critical regulator of early reprogramming initiation in mouse PSC. Indeed, upon initiation of somatic reprogramming, GCN5 coactivates Myc networks in PSC and coregulates a group of RNA splicing and RNA processing genes that are needed for somatic cell reprogramming [62]. In line with this evidence, GCN5 is required for the maintenance of histone acetylation in neural stem cells and cooperates with N-Myc to regulate overlapping transcriptional programs [63]. GCN5 also plays a key role in osteogenic commitment of mesenchymal stem cells (MSC) by inhibiting a nuclear factor kappa B-dependent transcription signalling pathway [64]. In osteogenic differentiation of periodontal ligament stem cells, GCN5 modulates DKK1, a central regulator of osteoblast activity. Mechanistically, GCN5 regulates DKK1 expression and promotes osteogenic differentiation by direct acetylation of lysine 9 and lysine 14 of H3 at the DKK1 promoter region [65]. Also, another member of the GNAT family, pCAF, plays a critical role in osteogenic differentiation of MSC, controlling bone morphogenetic protein gene expression by increasing H3K9Ac to their promoters [66].

The MYST family plays a crucial role in stem cells and development [67]. By using genome-wide chromatin immunoprecipitation sequencing and integrated transcriptome analyses, a recent study showed that the specific H4K16 acetyltransferase MOF is an integral component of the ESC core transcriptional network that plays an essential role in the maintenance of ESC self-renewal and pluripotency [68]. Further studies revealed that MOF is a crucial factor for efficient reprogramming of stem cells. Indeed, iPSCs express high levels of MOF, and this expression is dramatically upregulated following reprogramming. In addition, MOF depletion reduces H4K16Ac and H3K4me3 histone marks at the OCT4 promoter [69]. In ESCs, deletion of MOF determines an aberrant expression of NANOG, OCT4, and SOX2 [68]. A more recent study revealed a functional link among histone variants H3.3, MOF, and GLI1, which regulate neuronal SC proliferation and differentiation [70]. Tip60-deficient ESCs exhibited impaired differentiation into mesoderm and endoderm lineages, demonstrating a Tip60-dependent function in differentiation [71].

Several studies have also established the critical function of MOZ, in hematopoiesis [72, 73]. Notably, mice carrying mutation into the MOZ gene exhibit a defect to develop HSC during embryogenesis [74]. In both hematopoietic and neural stem cells, MOZ controls cell proliferation by repressing the transcriptional activity of p16(INK4a). Loss of MOZ determines the upregulation of p16(INK4a) in progenitor and stem cells and induces cell senescence, and depletion of p16(INK4a) reverts both the effects [75]. Intriguingly, despite the fact that MOF HAT activity is critical for hematopoietic cell maintenance, MOF is required for adult but not for early fetal hematopoiesis in mice [76].

### 3.3. Roles of HAT in Cancers

**Figure 1:**
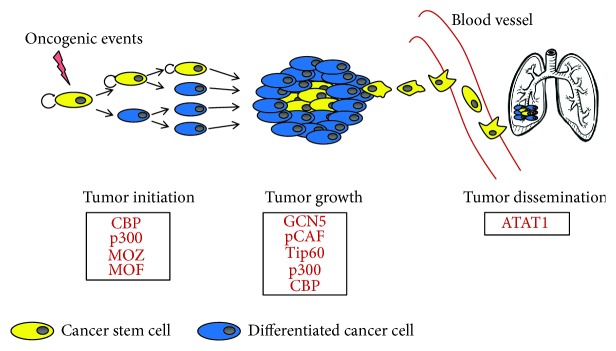
Schematic overview of HAT involved in cancer. HAT display critical roles in promoting different steps in cancer, starting from initiation and growth to dissemination towards target organs. CSCs play a key role in all these phases.

Genetic alterations and functional dysregulation of HAT are also strongly related to cancer [1]. In this context, it is well clear that HAT can have a dual function in carcinogenesis, acting as oncogenes or tumor suppressors. Different HAT members are reported to be mutated in tumors [27, 29, 77–79] and to be involved in different steps of tumor progression, starting from initiation and tumor growth to dissemination towards target organs (Figure 1).

Mutations of p300/CBP genes are associated with the development of different forms of leukemia and with B-cell non-Hodgkin's lymphoma [25, 79]. Mutations, which result in the truncation of the proteins, or deletion of p300/CBP genes have been also reported in different solid cancers, including lung, colon, breast, nasopharyngeal, ovarian, and cutaneous squamous cell carcinomas [78, 80–84].

Also, GNAT family members, for their cellular functions, have been implicated in different kinds of cancer. GCN5 is found to be upregulated in human glioma, colon, and lung cancer [85]. Conversely, the pCAF gene is frequently deleted in solid tumors such as ovarian, gastric, and esophageal carcinomas [86]. Recent reports suggest that also ATAT1 plays a key role in many cellular processes related to cancer dissemination, including cell adhesion, migration, and invasion [24, 87, 88]. Notably, ATAT1 is also associated with breast cancer progression [89].

Also, MYST family members have been often found mutated in cancer, and chromosomal aberrations involving different MYST genes and coding for hybrid proteins have been reported to drive leukemogenesis [90, 91]. HAT activity of MOF sustains a subtype of leukemia characterized by oncogenic rearrangements of the mixed-lineage leukemia (MLL) gene. Indeed, conditional deletion of MOF in a mouse model of MLL-AF9-driven leukemogenesis, and accordingly the treatment with small molecules targeting MYST members, reduces acute myeloid leukemia (AML) cell proliferation [76]. The expression of MOF has been often found altered also in solid cancers, such as breast, ovarian, renal, gastric, and colorectal carcinomas, as well as non-small-cell lung cancer and medulloblastoma [29]. The human Tip60 locus is frequently mutated or lost in a variety of tumors including breast and prostate carcinomas [92]. In the latter, prostate cancer Tip60 is upregulated and its expression correlates with disease progression [92]. Tip60 is also markedly increased in malignant mesothelioma compared to benign pleura, being overexpressed in both the sarcomatoid and biphasic subtypes [93].

### 3.4. Role of HAT in Cancer Stem Cells

For a long time, genetic alterations have been involved in cancer initiation and progression, but only in the past years, the role that chromatin modifications and epigenetic changes play in these processes emerges. It is possible that epigenetic changes in normal stem cells represent an early event of neoplastic transformation. On the other hand, also epigenetic alterations, and consequently the aberrant expression of a set of genes in more differentiated cells, may play a role in reprogramming into a pluripotent state and into an undifferentiated state. Indeed, the epigenetic changes in stem cells make these precursors susceptible to acquisition of mutations and give rise to tumor-initiating cells, also known as cancer stem cells (CSCs) [94]. CSCs for definition are a type of cancer cells that possess self-renewal capacity and the ability to differentiate into multiple cell types and are responsible for tumor initiation, recurrence, and drug resistance [95]. Developing functional assays identified CSCs as a cancer subpopulation with an *in vitro* self-renewal ability and an *in vivo* tumor-initiating and tumor-propagating ability.

The first experimental evidence for CSCs existence came from hematological malignancies, and then CSCs have been also identified in different solid tumors such as breast, lung, colon, and prostate carcinomas.

**Figure 2:**
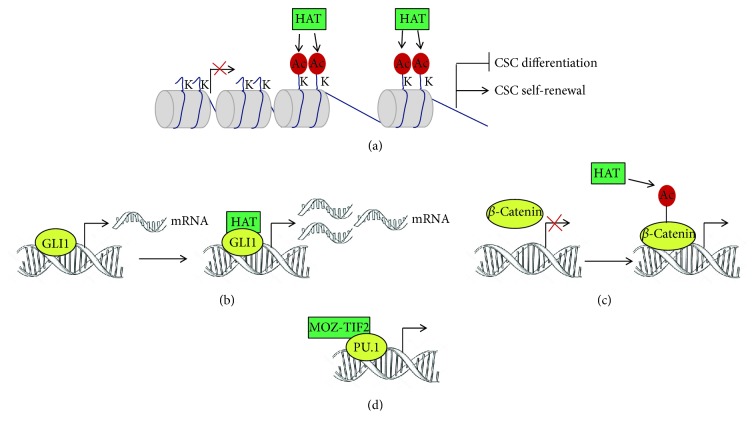
HAT regulate cancer stem cells through different mechanisms. HAT promote a cancer stem cell phenotype by inducing chromatin modifications (a), acting as a transcriptional coactivator (b) or acetylating cancer stem cell transcription factors (c). HAT can also constitute chimeric proteins as a result of chromosome translocation (d), which possess aberrant HAT activities.

The analysis of the epigenetic factors implicated in the regulation of CSCs self-renewal has been hampered by experimental and technical challenges, strictly related to the difficulty of *in vitro* isolation and expansion of CSCs from solid tumors. This is probably the reason why the first evidence of a link between CSCs and HAT has been obtained in hematological tumors [96, 97]. Indeed, HAT affect CSCs properties (1) by inducing chromatin modifications, (2) by acting as transcriptional coactivators, or (3) by acetylating CSCs transcription factors (Figure 2).

Several studies revealed that p300 is a coactivator of Myb, a transcription factor essential for the proliferation of hematopoietic cells, and that targeting this interaction may have therapeutic potential for the AML treatment [98]. Accordingly, the role for CBP and p300 in the induction and maintenance of AML has been described [99, 100], and targeting CBP/p300 HAT activity by small molecules shows preclinical efficacy in different AML subtypes [101].

It is not surprising that the signalling pathways playing an important role in stemness and differentiation program of ESCs (i.e., Wnt/*β*-catenin, Notch, Hedgehog, TGF*β*/BMP, JAK/Stat, and Hippo) may also contribute to CSCs maintenance and that HAT or protein acetylation may be a critical regulator of these pathways. In this scenario, p300 and CBP act as important coactivators of the Wnt/*β*-catenin pathway also in CSCs [102]. For instance, in uterine sarcomas, the *β*-catenin/p300 signal pathway cooperates with SOX genes to promote Slug expression during divergent sarcomatous differentiation in uterine carcinosarcoma [103]. In nasopharyngeal carcinoma cells, the targeting of the interaction of CBP/*β*-catenin impairs the cancer stem-like population reducing the expression of CSCs-associated markers [104]. Notably, also ATAT1 is associated with pancreatic cancer-initiating cells [89].

The role of pCAF in the control of Hedgehog signalling, a master regulator of tissue development, stemness, and tumorigenesis, has been also described. pCAF forms a complex with GLI1 on target promoters, thus enhancing transcription by promoting H3K9Ac at Hedgehog/GLI1 target gene promoters [105]. According to these papers highlighting the role of this family in CSCs, the inhibition of GNAT members by the small molecule named CPTH6 [106, 107] induces apoptosis in lung CSCs derived from lung cancer patients and its growth inhibitory effect is correlated with the baseline level of K40-acetylated *α*-tubulin [108].

As mentioned above, chromosome translocations of MYST members lead to the generation of chimeric proteins, such as MOZ-CBP, MOZ-p300, MORF-CBP, and MOZ-TIF2, which possess aberrant HAT activity [29]. Among these hybrid proteins, MOZ-TIF2 has been shown to promote self-renewal of leukemic stem cells. Mechanistically, the interaction between MOZ-TIF2 and the transcription factor PU.1 stimulates the expression of the macrophage colony-stimulating factor receptor [97], a factor required for self-renewal of leukemic stem cells.

## 4. Conclusion

The dynamic changes of acetylation in histone and nonhistone proteins could affect their functions in several biological processes and further lead to different kinds of diseases. Recent advances have demonstrated that protein acetylation plays important roles in the proliferation and differentiation of both normal and cancer cells but the regulatory pathways involved in the acetylation state of malignant cells are still not completely elucidated. Considering the critical role of CSCs in the pathogenesis of cancer, targeting acetylation could represent a promising strategy for the treatment of different malignancies. In this context, the relevance of HAT inhibitors for cancer therapy is worthy of study and needs more investigations in the future. Ultimately, deeper investigation of the histone acetylation pattern in different cancer histotypes and its regulation are needed to better appreciate the link of acetylation deregulation and cancer, and to develop more efficient anticancer approaches.

## Figures and Tables

**Table 1:** HAT classification.

Nomenclature	Cellular localization	Histone and nonhistone
*Type A HAT*		
*p300/CBP family*		
CBP (KAT3A)	Nucleus	NF-kappaB, c-myb, Foxo1, NCOA3, PCNA, KLF1, transcription factor MafG, IRF-2
p300 (KAT3B)	Nucleus	H2A, H2B, H3, NF-kappaB, c-myc, p53, STAT3, *β*-catenin, Foxo1, AR, ALX1, SIRT2, HDAC1, BCL6, MTA1, XBP1 isoform 2, PCNA, MEF2D, ZBTB7B
*GNAT family*		
KAT2		H3, H2B
GCN5 (KAT2A)	Nucleus	H3, H4, H2A, CDC6, CDK9,cyclin D1, cyclin E1 and E2F1, HDM2, PTEN, c-myc, TBX5, PLK4, CEBPB
pCAF (KAT2B)	Nucleus	H3, H4, p53, CDK9, c-myc, Foxo1, AR, TBX5, PLK4, ACLY
ELP3 (KAT9)		H4, H2A, H3
ATAT-1 (MEC-17)	Cytosol	*α*-Tubulin, cortactin
*MYST family*		
Tip60 (KAT5)	Nucleus	H4, H2A, ATM, TRRAP, E2F1, c-myc, NR1D2, FOXP3, RAN
KAT6		
MOZ (KAT6A)	Nucleus	H3, p53
MORF (KAT6B)	Nucleus	
HBO1 (KAT7)	Nucleus	H3, H4
MOF (KAT8)	Nucleus	H4, p53
*Transcription factor complexes*		
TAF1/TBP (KAT4)	Nucleus	H3 H4
TFIIIC90 (KAT12)	Nucleus	H3
*Nuclear receptor coactivators*		
SCR1 (KAT13)	Nucleus	H3 H4
*Camello family*		
Camello	Perinucleus	H4
*Type B HAT*		
HAT1 (KAT1)	Nucleus/cytosol	H3, H4, H2A
HAT4 (KAT4)	Cytosol	H4 H2A

**Table 2:** Involvement of HAT in normal development.

HAT	Organism	Impact on development	Ref
CBP	Null mice	Neural tube closure and embryonic vascular and cardiac defects	[35]

CBP	Mice harbouring point mutation or deletion of the HAT domain	Several defects in memory and synaptic plasticity	[37, 38]

CBP	Null mice	Rubinstein-Taybi syndrome (RTS) multilineage	[8]

p300	Heterozygous mice	Embryonic lethality	[35]

p300	Mice harbouring point mutation	Defects in the hematopoiesis (B-cell deficiency, megakaryocytosis, and thrombocytosis)	[39]

GCN5	Mice harbouring a point mutations abrogating GN5 HAT activity	Cranial neural tube closure defects and exencephaly	[41]

GCN5	Null mice	Early embryonic lethality	[40]
	Knockdown zebrafish	Cardiac, fin, and limb defects	[45]

pCAF	Null mice	Normal phenotype in the embryo	[42, 44]
		Defects in learning abilities and short-term and long-term memory in adult	[44]
	Knockdown zebrafish	Cardiac, fin, and limb defects	[45]

ATAT-1	Null mice	Viable, without morphological defects; loss of *α*-tubulin acetylation in sperm flagella; dentate gyrus distortion	[46]

**Table 3:** Involvement of HAT in stem cells.

HAT	Target	Effect in normal stem cells	Ref
CBP		Maintenance of self-renewal hematopoietic stem cells	[61]

p300	OCT4, SOX2, KLF4	Stem cell reprogramming	[54]
		Proliferation and odontogenic differentiation of human dental pulp cells	[56]
		Self-renewal and pluripotency maintenance in ESCs	[57]
		Proper hematopoietic differentiation	[61]

GCN5	Myc	Early reprogramming initiation in mouse PSC	[62]
	NF-*κ*B	Osteogenic commitment of mesenchymal stem cells	[64]
	DKK1	Osteogenic differentiation of periodontal ligament stem cells	[65]

pCAF	BMPs	Osteogenic differentiation of mesenchymal stem cells	[66]

MOF	NANOG, OCT4, SOX2	Maintenance of ESC self-renewal and pluripotency	[68]
		Stem cell reprogramming	[69]

Tip60		ESC differentiation into mesoderm and endoderm lineages	[71]

MOZ	p16(INK4a)	Maintenance of proliferation in hematopoietic and neural stem cells	[72]
